# Responses of perivascular macrophages to circulating lipopolysaccharides in the subfornical organ with special reference to endotoxin tolerance

**DOI:** 10.1186/s12974-019-1431-6

**Published:** 2019-02-14

**Authors:** Shoko Morita-Takemura, Kazuki Nakahara, Sanae Hasegawa-Ishii, Ayami Isonishi, Kouko Tatsumi, Hiroaki Okuda, Tatsuhide Tanaka, Masahiro Kitabatake, Toshihiro Ito, Akio Wanaka

**Affiliations:** 1 Department of Anatomy and Neuroscience, Faculty of Medicine, Nara Medical University, 840 Shijo-cho, Kashihara, Nara 634-8521 Japan; 2 Faculty of Health Sciences, Kyorin University, Tokyo, Japan; 3 Department of Anatomy, Graduate School of Medical Science, Kanazawa University, Kanazawa, Japan; 4 Department of Immunology, Nara Medical University, Kashihara, Nara Japan

**Keywords:** Bone marrow-derived cells, Endotoxin tolerance, Interleukin-1β, Macrophage depletion, Sickness behavior

## Abstract

**Background:**

Circulating endotoxins including lipopolysaccharides (LPS) cause brain responses such as fever and decrease of food and water intake, while pre-injection of endotoxins attenuates these responses. This phenomenon is called endotoxin tolerance, but the mechanisms underlying it remain unclear. The subfornical organ (SFO) rapidly produces proinflammatory cytokines including interleukin-1β (IL-1β) in response to peripherally injected LPS, and repeated LPS injection attenuates IL-1β production in the SFO, indicating that the SFO is involved in endotoxin tolerance. The purpose of this study is to investigate features of the IL-1β source cells in the SFO of LPS-non-tolerant and LPS-tolerant mice.

**Methods:**

We first established the endotoxin-tolerant mouse model by injecting LPS into adult male mice (C57BL/6J). Immunohistochemistry was performed to characterize IL-1β-expressing cells, which were perivascular macrophages in the SFO. We depleted perivascular macrophages using clodronate liposomes to confirm the contribution of IL-1β production. To assess the effect of LPS pre-injection on perivascular macrophages, we transferred bone marrow-derived cells obtained from male mice (C57BL/6-Tg (CAG-EGFP)) to male recipient mice (C57BL/6N). Finally, we examined the effect of a second LPS injection on IL-1β expression in the SFO perivascular macrophages.

**Results:**

We report that perivascular macrophages but not parenchymal microglia rapidly produced the proinflammatory cytokine IL-1β in response to LPS. We found that peripherally injected LPS localized in the SFO perivascular space. Depletion of macrophages by injection of clodronate liposomes attenuated LPS-induced IL-1β expression in the SFO. When tolerance developed to LPS-induced sickness behavior in mice, the SFO perivascular macrophages ceased producing IL-1β, although bone marrow-derived perivascular macrophages increased in number in the SFO and peripherally injected LPS reached the SFO perivascular space.

**Conclusions:**

The current data indicate that perivascular macrophages enable the SFO to produce IL-1β in response to circulating LPS and that its hyporesponsiveness may be the cause of endotoxin tolerance.

**Electronic supplementary material:**

The online version of this article (10.1186/s12974-019-1431-6) contains supplementary material, which is available to authorized users.

## Background

Circulating endotoxins, including lipopolysaccharides (LPS), induce fever, reduce locomotor activity, and decrease food and water intake [1]. These behavioral changes are commonly referred to as sickness behavior, which is uncomfortable but important to fight infection. However, pre-injection of LPS attenuates sickness behavior after subsequent challenge with LPS [2, 3]. Since this phenomenon, known as endotoxin tolerance, can occur naturally in humans and is an important system for host protection against excessive response to endotoxin and endotoxin shock, clarifying the mechanisms for development of tolerance to endotoxins is needed.

Sickness behavior is due to the effects of proinflammatory cytokines on the brain [4]. Although circulating immune cells produce proinflammatory cytokines following infection, endotoxin-induced sickness behavior sometimes precedes the increase in plasma cytokine concentration (for reviews, see [4, 5]). These data raise the possibility that the brain communicates directly with circulating endotoxins. However, the blood-brain barrier (BBB) prevents circulating endotoxins from freely entering the brain.

Accumulating evidence indicates that the subfornical organ (SFO) is a brain area that directly senses circulating LPS to produce proinflammatory cytokines. A sublethal dose of LPS causes a rapid production of proinflammatory cytokines including interleukin-1β (IL-1β), interleukin-6, and tumor necrosis factor-α in the SFO [6, 7]. Among these cytokines, IL-1β is important for the induction of sickness behavior (for a review, see [8]). Local injection of an IL-1 receptor antagonist into the SFO attenuates LPS-induced fever [9]. These data suggest that the SFO directly senses circulating LPS and produces IL-1β to induce sickness behavior. Moreover, LPS-induced IL-1β production disappeared in the SFO of LPS-pre-injected rabbits [7]. The attenuation of IL-1β expression suggests that the SFO is involved in endotoxin tolerance. However, what cells produce IL-1β in response to circulating LPS and why the SFO ceases to produce IL-1β in LPS-pre-injected animals remain unclear.

Circulating molecules including LPS are thought to diffuse freely into the SFO, since the SFO is a specialized brain area that lacks a typical BBB. However, we have recently shown that peripherally injected tracers (MW > 10,000) accumulate around the SFO vasculature [10, 11]. In the SFO, the perivascular basement membranes are separated into the parenchymal cell-derived outer basement membrane and the endothelial cell-derived inner basement membrane and create a large perivascular space outside the brain parenchyma [12]. Our peripherally injected tracers localized in the perivascular space in the SFO. Since LPS tend to form micelles (MW ~ 300,000–1000,000), with hydrophilic polysaccharides facing outward [13], it is unlikely that circulating LPS simply diffuse into the SFO. In addition, macrophages around the SFO vasculature were proposed as IL-1β source cells [9]. These data raise the possibility that macrophages in the SFO perivascular space are responsible for IL-1β production in response to circulating LPS and participate in endotoxin tolerance.

Although microglia are brain-resident myeloid cells, macrophages also exist in the perivascular space, meninges, choroid plexus, and circumventricular organs including the SFO [14]. The resident microglia arise from the yolk sac and populate the neuroepithelium in early embryogenesis [15], whereas the SFO macrophages are replenished by bone marrow-derived cells [16, 17].

Here, we investigated features of the SFO macrophages and their production of LPS-induced IL-1β in LPS-non-tolerant and LPS-tolerant mice, using macrophage depletion and bone marrow transplantation methods.

## Methods

### Animal preparation

Eight-week-old male mice (C57BL/6J) were purchased from CLEA Japan. (Tokyo, Japan). Male C57BL/6-Tg (CAG-EGFP) mice and C57BL/6N mice were purchased from Japan SLC (Hamamatsu, Japan). In C57BL/6-Tg (CAG-EGFP) mice, ubiquitously active cytomegalovirus immediate early enhancer-chicken β-actin hybrid (CAG) promoter drives enhanced green fluorescent protein (EGFP) expression. The animals were housed in a colony room with a 12-h light/12-h dark cycle, given access to commercial chow (CE-2, CLEA Japan) and tap water ad libitum, and maintained under pathogen-free conditions. We measured food intake by subtracting the weight of the food container and taking into account any spillage, water consumption by measuring the weight of the water bottle (Shin Factory, Fukuoka, Japan), and body weight daily between 09:00 and 10:00. Animal care and the experiments were conducted in accordance with the National Institutes of Health Guide for the Care and Use of Laboratory Animals and the Guidelines for Proper Conduct of Animal Experiments of the Science Council of Japan. The Animal Care Committee of Nara Medical University approved the experimental protocol.

### Administration of LPS

Mice were intraperitoneally injected with either LPS (1 mg/kg) from *Escherichia coli* (serotype O55:B5, Sigma-Aldrich Japan, Tokyo, Japan) or vehicle (pyrogen-free physiological saline, Ohtsuka Chemical, Tokushima, Japan) as previously described [14]. To examine the effect of LPS pre-injection, LPS were again injected 2 or 4 days after the first LPS injection.

### Administration of fluorescent tracers

Mice were subjected to intravenous (caudal vein) injection (100 μl) of Texas Red-conjugated lysine-fixable Dextran 70,000 (MW: 70,000, Molecular Probes, 20 mg/ml) in phosphate-buffered saline (PBS; pH 7.4). Animals were sacrificed at 30 min after this injection for immunohistochemical analysis.

### Administration of clodronate liposomes

To label control empty liposomes, liposomes were preincubated with 0.125 mg/ml of DiI (Wako, Osaka, Japan) for 10 min and centrifuged (20,000*g*) for 10 min three times with resuspension in sterile PBS to wash out unlabeled DiI. Mice were subjected to intravenous (caudal vein) injection (200 μl) of DiI-labeled control empty liposomes in PBS. Animals were sacrificed at 1 h after this injection for immunohistochemical analysis.

To investigate the contribution of perivascular macrophages to LPS-induced IL-1β expression in the SFO, mice were subjected to intravenous (caudal vein) injection (200 μl) of clodronate liposomes or control empty liposomes (Macrokiller V100, Cosmo Bio, Tokyo, Japan) in PBS. Three days after the liposome injection, mice were intraperitoneally injected with LPS (1 mg/kg). Animals were sacrificed at 1 h after LPS injection for immunohistochemical analysis.

### Preparation of bone marrow chimeras

We employed two different immunosuppression methods: (1) irradiation with head shielding or (2) busulfan treatment, as previously reported [15–17]. Previously, bone marrow-derived cells have been found in the SFO following bone marrow transplantation into whole-body-irradiated mice [16, 17]. Although most bone marrow transplantation experiments have used whole-body irradiation, this method may perturb the integrity of the BBB [17]. These two immunosuppression methods were selected to avoid the artificial effects of whole-body irradiation. (1) Mice received irradiation (6 Gy × 2 with a 4-h interval) 24 h prior to bone marrow reconstitution using an X-ray irradiator (Model MBR-1520R, Hitachi Power Solutions, Ibaraki, Japan). Mice were exposed to X-rays with 4-mm-thick individual lead shields positioned to protect the head and to irradiate only the lower part of the body. (2) Mice were intraperitoneally injected with the chemotherapeutic agent busulfan (30 μg/g body weight; B2635, Sigma-Aldrich Japan) in a 1:4 solution of dimethyl sulfoxide and PBS 7, 5, and 3 days prior to bone marrow transfer.

All mice were treated with antibiotics (trimethoprim/sulfamethoxazole) for 14 days after irradiation or busulfan treatment. Bone marrow-derived cells were obtained from the femur and tibia of 5-week-old male C57BL/6-Tg (CAG-EGFP) mice and resuspended in PBS with 2% fetal bovine serum. Bone marrow-derived cells (1 × 10^6^) were transferred to 8-week-old male C57BL/6N recipient mice by tail vein injection (100 μl). Mice were left undisturbed for 1 month to allow engraftment. For quantitative analysis, engraftment was verified by determining the percentage of EGFP-expressing cells in the blood. We counted the numbers of EGFP^+^ cells in peripheral blood by flow cytometry and confirmed efficient chimerism as demonstrated by the large proportions of circulating blood leukocytes expressing EGFP (range between 62.7 and 73.7%).

We found EGFP^+^ Iba1^+^ perivascular macrophages in the SFO both of irradiated and busulfan-treated mice (Additional file 1: Figure S1). There were many more EGFP^+^ Iba1^+^ cells in the SFO of busulfan-treated mice than of irradiated mice. We therefore employed busulfan for immunosuppression in Fig. 4.

### Light microscopic immunohistochemistry

Mice were transcardially perfused with PBS followed by 4% paraformaldehyde in 0.1 M phosphate buffer (pH 7.4) under deep pentobarbital anesthesia. The dissected brains were postfixed for 6 h, cryo-protected with 30% sucrose in PBS, and frozen at − 80 °C in Tissue-Tek OCT compound (Sakura Finetechnical, Tokyo, Japan). Coronal sections were cut at a thickness of 30 μm with a cryostat (Leica, Heidelberg, Germany) at − 15 °C. For immunofluorescence detection, we processed free-floating sections as described previously [18]. In brief, sections were washed with PBS and treated with 25 mM glycine in PBS for 20 min. When mouse primary antibodies were used, the sections were further treated with unlabeled goat Fab against mouse IgG (Jackson ImmunoResearch Laboratories, West Grove, PA, USA; 1:400) for 2 h to mask endogenous mouse IgG-like proteins. They were then incubated with 5% normal donkey serum in PBS containing 0.3% Triton X-100 for 1 h and then with mouse IgG against desmin (clone D33, DAKO, Glostrup, Denmark; 1:800), against glial fibrillary acidic protein (GFAP; clone GA5, Cell Signaling Technology, Danvers, MA, USA; 1:1000), and against LPS (clone 2D7/1, abcam, Cambridge, UK; 1:200); goat IgG against CD206 (R&D Systems, Minneapolis, MN, USA; 1:1000) and against IL-1β (AF-401-NA, R&D Systems; 1:1000); and rabbit IgG against ionized calcium-binding adaptor molecule 1 (Iba1, Wako; 1:500) for 24 h at 4 °C; and with guinea pig IgG against laminin-111 (the antigen was laminin-111 from Engellbreth-Holm-Swarm murine sarcoma basement membrane; kindly gifted by S. Miyata, Kyoto Institute of Technology, Kyoto, Japan [19]; 1:200) for 2 h at 37 °C). The primary antibodies were visualized by incubating the sections in Alexa 405-, 488-, 594-, and 633-conjugated donkey IgG against goat, rabbit, rat, and guinea pig (Jackson ImmunoResearch Laboratories; 1:1000) in PBS containing 0.3% Triton X-100 for 1 h. When we used mouse IgG primary antibodies, Alexa 488- and 594-conjugated goat F(ab)_2_ against mouse IgG (Jackson ImmunoResearch Laboratories; 1:500) was used to avoid nonspecific binding of endogenous mouse Fc receptors. Nuclei were counterstained with 4,6-diamidino-2-phenylindole.

For LPS immunohistochemistry, we performed antigen retrieval by incubating sections in citrate-ethylenediaminetetraacetic acid buffer (10 mM citric acid, 2 mM ethylenediaminetetraacetic acid, 0.05% Tween 20, pH 6.2) for 20 min at 95 °C. For CD206 immunohistochemistry, the sections were pretreated with chilled acetone-methanol for 15 min.

In order to determine IL-1β antibody specificity, the primary antibody was preincubated with 4 μg/ml of the mouse IL-1β recombinant protein (401-ML, R&D Systems) for 24 h at 4 °C. IL-1β immunoreactivity was abolished by primary antibody preadsorption in the SFO of both vehicle- and LPS-injected mice (Additional file 1: Figure S2).

### Confocal microscopic observation

Labeled sections were mounted on glass slides, and coverslips were sealed with Vectashield (Vector Laboratories, Burlingame, CA, USA). We observed stained sections using a laser-scanning confocal microscope (C2, Nikon, Tokyo, Japan) by changing the fine focus of the microscope for quantitative analysis. Single-plane images were acquired to make figures using a × 20 objective with 0.75 NA, × 40 objective with 0.95 NA, or × 60 water immersion objective with 1.20 NA at a 1024 × 1024 pixel resolution. We saved images as TIF files using Nikon Nis-Elements AR software and arranged them using Photoshop CS6.

### Data analysis

All statistical analyses were carried out in SPSS (Version 23.0, IBM SPSS Statistics, Ehningen, Germany).

Statistical comparisons for food intake, water intake, and body weight were performed using one-way ANOVA with Scheffé’s post hoc test. Difference was assessed at a significance level of *P* < 0.05. Data are shown as mean ± SEM.

For quantitative analyses of cell numbers, analysis of all images was performed such that the experimenter was blind to the treatment group. We used four animals from each group for quantification, and at least eight sections per animal were chosen from the SFO region (between bregma − 0.23 and − 0.71 mm) according to the mouse brain atlas [20]. Area measurement was performed by delineating manually each SFO using ImageJ software (National Institutes of Health). Independent groups were compared with Student’s *t* test or one-way ANOVA with Scheffé’s post hoc test. Difference was assessed at a significance level of *P* < 0.05. Data are shown as mean ± SEM.

## Results

### Mice develop endotoxin tolerance

**Fig. 1:**
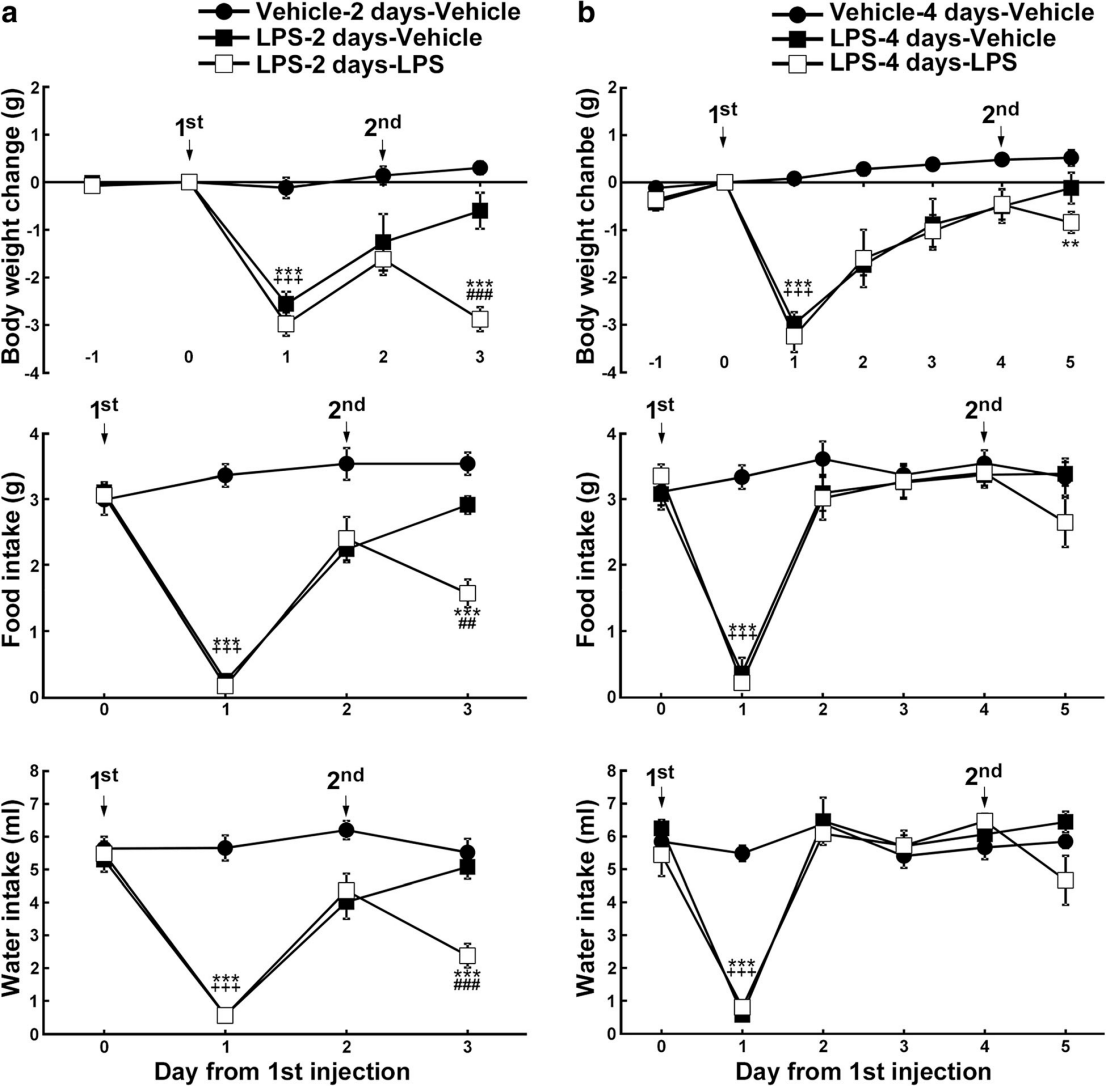
Effects of LPS injections on body weight, food intake, and water intake. The first injection of vehicle or LPS was given on day 0 and the second on days 2 (**a**) or 4 (**b**). Vehicle-2 (4) days-vehicle, mice were injected intraperitoneally with saline for the first and second injections; LPS-2 (4) days-vehicle, mice were injected intraperitoneally with LPS for the first injection and saline for the second injection; LPS-2 (4) days-LPS, mice were injected intraperitoneally with LPS for the first and second injections. *N* = 5. **P < 0.01, ***P < 0.001 LPS-2 (4) days-LPS vs vehicle-2 (4) days-vehicle, ^##^*P* < 0.01, ^###^*P* < 0.001 LPS-2 (4) days-LPS vs LPS-2 (4) days-vehicle, ^+++^*P* < 0.001 LPS-2 (4) days-vehicle vs vehicle-2 (4) days-vehicle, determined by one-way ANOVA with Scheffé’s post hoc test

We first examined the effect of LPS pre-injection on endotoxin tolerance. We measured LPS-induced sickness behavior by assessing changes in body weight and in food and water consumption at 24 h post injection, since LPS-induced fever is accompanied by a decrease of body weight and a reduction of food and water intake in the mice, and second LPS-induced tolerance is observed for these indexes [1]. In addition, the SFO is involved in the regulation of food and water intake [21, 22]. When the second LPS injection was given 2 days after the first injection (LPS-2 days-LPS), body weight and food and water intake were significantly reduced after the second LPS injection (Fig. 1a). On the other hand, mice did not exhibit significant reduction in body weight and food or water consumption after the second LPS injection given 4 days after the first injection (LPS-4 days-LPS), with the exception that body weight was reduced in LPS-4 days-LPS mice compared to vehicle-4 days-vehicle mice (*F*_2,12_ = 7.071, *P* = 0.009) (Fig. 1b). These data indicate that mice developed endotoxin tolerance 4 days after the first LPS injection.

### Perivascular macrophages are the source of IL-1β in the SFO after the first LPS injection

**Fig. 2:**
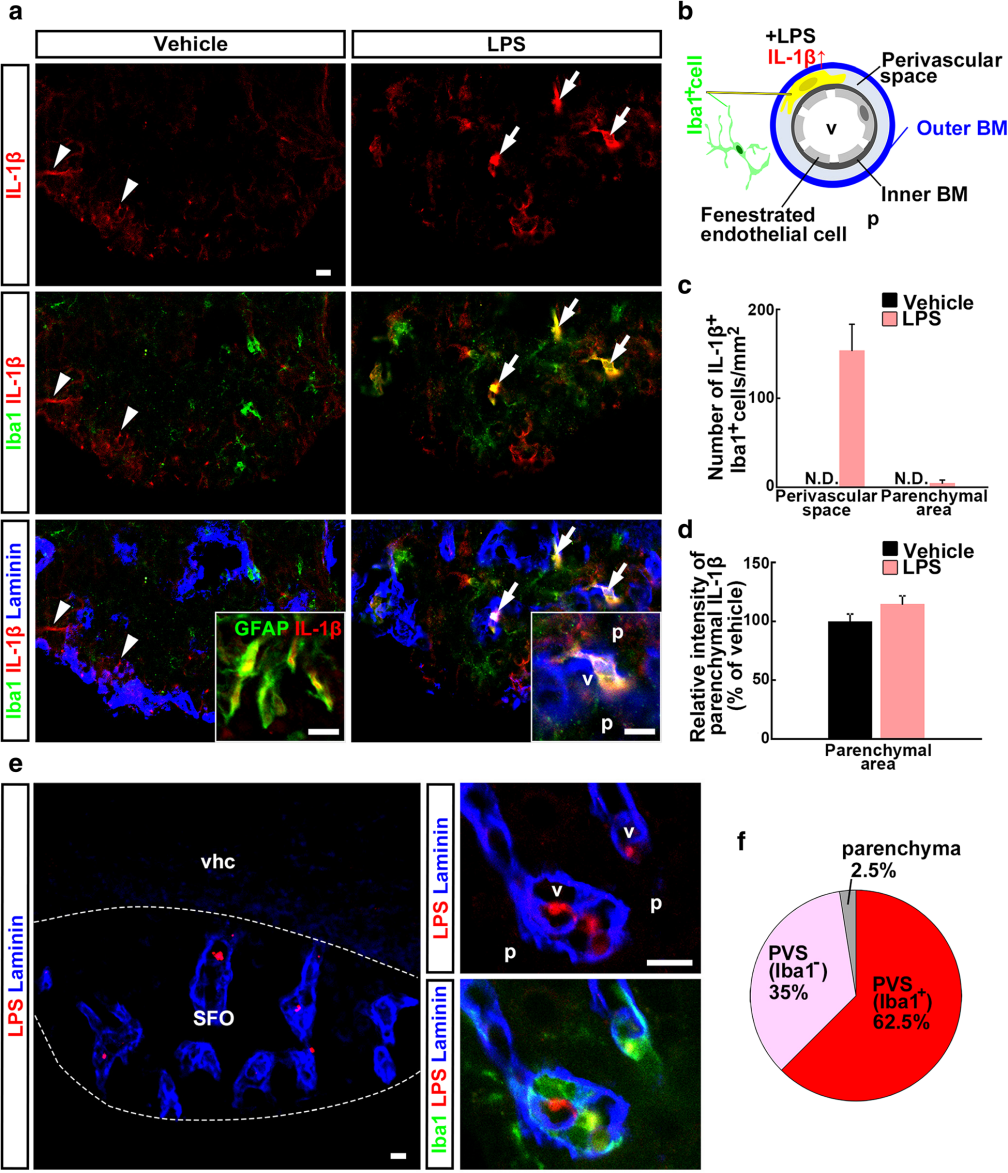
Effect of LPS injection on IL-1β immunoreactivity in the SFO. Cryosections were immunostained with antibodies against IL-1β, the myeloid lineage cell marker Iba1, the outer basement membrane marker laminin-111, the astrocyte marker GFAP, and LPS. **a** IL-1β immunoreactivity (arrowheads) was found as processes and co-localized with GFAP^+^ astrocytes in the parenchyma of the SFO of vehicle-injected control mice. Strong IL-1β immunoreactivity (arrows) was seen in the SFO following LPS injection. IL-1β immunoreactivity was observed in Iba1^+^ cells situated inside the laminin-111^+^ outer basement membrane. **b** Summary diagram. **c** Quantitative analysis revealing the effect of LPS on the number of IL-1β^+^ Iba1^+^ cells in the SFO of adult mice. *N* = 4. **d** Quantitative analysis revealing the effect of LPS on the relative intensity of parenchymal IL-1β immunoreactivity in the SFO of adult mice. *N* = 4. **e** Immunoreactivity of LPS was found in the SFO, but not in the adjacent ventral hippocampal commissure. Immunoreactivity of LPS was found in the perivascular space and sometimes co-localized with Iba1 immunoreactivity. **f** Quantification of results from **e**. Data were obtained from 4 animals (38 sections) and 80 LPS^+^ spots were examined. Laminin, laminin-111; N.D., not detected; p, parenchymal area; PVS, perivascular space; v, vasculature; vhc, ventral hippocampal commissure. Scale bars are 10 μm

Next, we sought to identify the IL-1β source cells in the SFO after the first LPS injection. We observed moderate IL-1β immunoreactivity in GFAP^+^ astrocytes in the SFO of vehicle-injected control mice (Fig. 2a). One hour after LPS injection, enhanced expression of IL-1β was observed in the SFO. Enhanced immunoreactivity of IL-1β co-localized with that of some myeloid lineage cell marker Iba1^+^ cells and was often observed inside the laminin-111^+^ basement membrane. IL-1β was mainly expressed in the Iba1^+^ cells that exist inside, but sometimes spanning, the laminin-111^+^ vascular outer basement membrane that clarifies the border between the perivascular space and parenchyma (154.1 ± 29.1 cells/mm^2^), and rarely expressed in the Iba1^+^ parenchymal cells that exist outside this membrane (4.9 ± 3.3 cells/mm^2^) (Fig. 2a–c). Relative intensity of parenchymal IL-1β in LPS-injected mice was not significantly different from that in vehicle-injected mice (Fig. 2d).

We then investigated the Iba1^+^ cells in the perivascular space. Typical perivascular macrophages are elongated or ameboid-like, express mannose receptor CD206 and low levels of Iba1, and are endowed with phagocytic activity [23]. In the SFO, morphologies of perivascular Iba1^+^ cells were elongated or ameboid-like, whereas parenchymal Iba1^+^ microglia were ramified (Additional file 1: Figure S3a). Both the SFO perivascular Iba1^+^ cells and typical perivascular macrophages expressed CD206 (Additional file 1: Figure S3b). The SFO perivascular Iba1^+^ cells strongly expressed Iba1, whereas typical brain perivascular macrophages expressed lower levels of Iba1 than microglia, consistent with a previous report [23]. The fluorescence of peripherally injected tracer Dextran 70,000 was often localized in the SFO perivascular Iba1^+^ cells as numerous puncta (Additional file 1: Figure S3c, d), meaning that these cells phagocytose peripherally injected molecules. Since brain pericytes can express Iba1 following ischemia [24], we examined the expression patterns of Iba1 and the pericyte marker desmin. However, perivascular Iba1^+^ cells did not overlap with desmin^+^ pericytes in the SFO (Additional file 1: Figure S3e). These data indicate that perivascular Iba1^+^ cells in the SFO were perivascular macrophages.

To explore why perivascular macrophages expressed IL-β but parenchymal microglia did not, we examined the distribution of peripherally injected LPS in the SFO. We found that immunoreactivity of peripherally injected LPS localized inside the laminin-111^+^ outer basement membrane in the SFO, sometimes co-localizing with immunoreactivity of Iba1 (Fig. 2e). The majority of LPS^+^ spots were observed in the perivascular space (Fig. 2f; 97.5%).

**Fig. 3:**
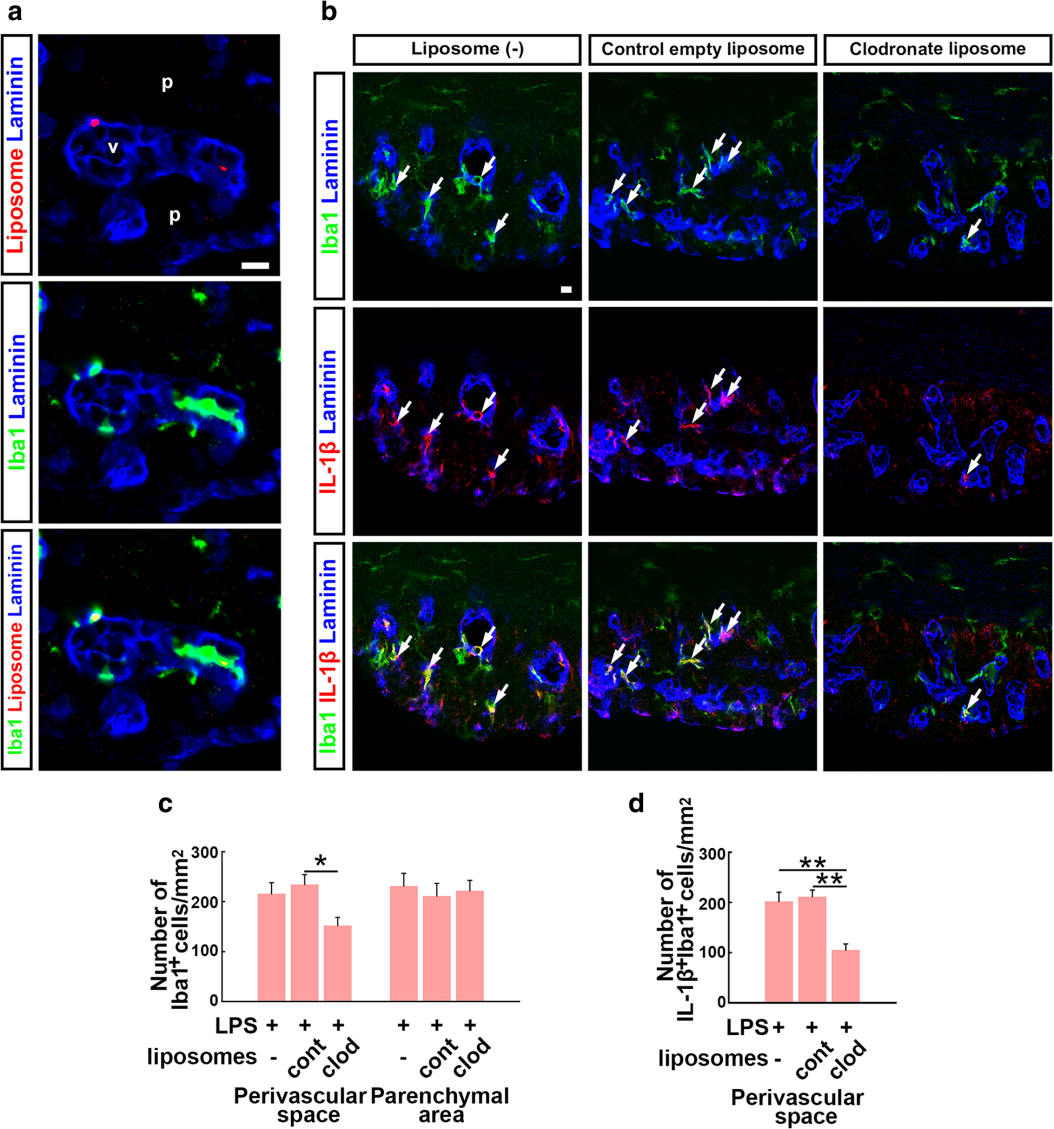
Effect of macrophage deprivation on LPS-induced IL-1β immunoreactivity in the SFO. Cryosections were immunostained with antibodies against the outer basement membrane marker laminin-111, the myeloid lineage cell marker Iba1, and IL-1β. **a** Localization of peripherally injected control liposomes in the SFO. DiI-labeled control liposomes were found in the SFO perivascular space and co-localized with Iba1 immunoreactivity. **b** IL-1β immunoreactivity was found in the perivascular space (arrows) of the SFO following LPS injection in liposome (−) mice and control empty liposome-injected mice. A decreased number of Iba1^+^ cells and attenuated IL-1β immunoreactivity were seen in the perivascular space of the SFO following LPS injection in clodronate liposome-injected mice. **c** Quantitative analysis revealing the effects of clodronate liposome injection on the number of Iba1^+^ cells in the SFO of adult mice. *N* = 4. *P < 0.05, determined by one-way ANOVA with Scheffé’s post hoc test. **d** Quantitative analysis revealing the effects of clodronate liposome injection on the number of IL-1β^+^ Iba1^+^ cells in the perivascular space of the adult mice SFO. *N* = 4. **P < 0.01, determined by one-way ANOVA with Scheffé’s post hoc test. clod, clodronate liposomes; cont, control empty liposomes; Laminin, laminin-111; p, parenchymal area; v, vasculature. Scale bars are 10 μm

To confirm the contribution of perivascular macrophages to LPS-induced IL-1β expression in the SFO, macrophages were depleted using clodronate liposomes. The phagocytosis-mediated uptake of clodronate leads to apoptosis and abrogation of macrophage functions [25]. We found that DiI-labeled control liposomes localized inside the laminin-111^+^ outer basement membrane in the SFO and co-localized with immunoreactivity of Iba1 (Fig. 3a). We observed co-localization of DiI-labeled control liposomes and immunoreactivity of Iba1 in the liver as positive control (Additional file 1: Figure S4). Clodronate liposome injection significantly decreased the number of Iba1^+^ cells (*F*_2,9_ = 4.909, *P* = 0.045 as compared to control empty liposome injection), and the number of IL-1β^+^ Iba1^+^ cells (*F*_2,9_ = 15.519, *P* = 0.004 as compared to without liposome injection and *P* = 0.002 as compared to control empty liposome injection) in the SFO perivascular space after LPS injection (Fig. 3b–d). These data indicate that circulating LPS localized in the SFO perivascular space and that perivascular macrophages were the source of IL-1β in the SFO after the first LPS injection.

### Bone marrow-derived perivascular macrophages in the SFO increase in number in endotoxin-tolerant mice

**Fig. 4:**
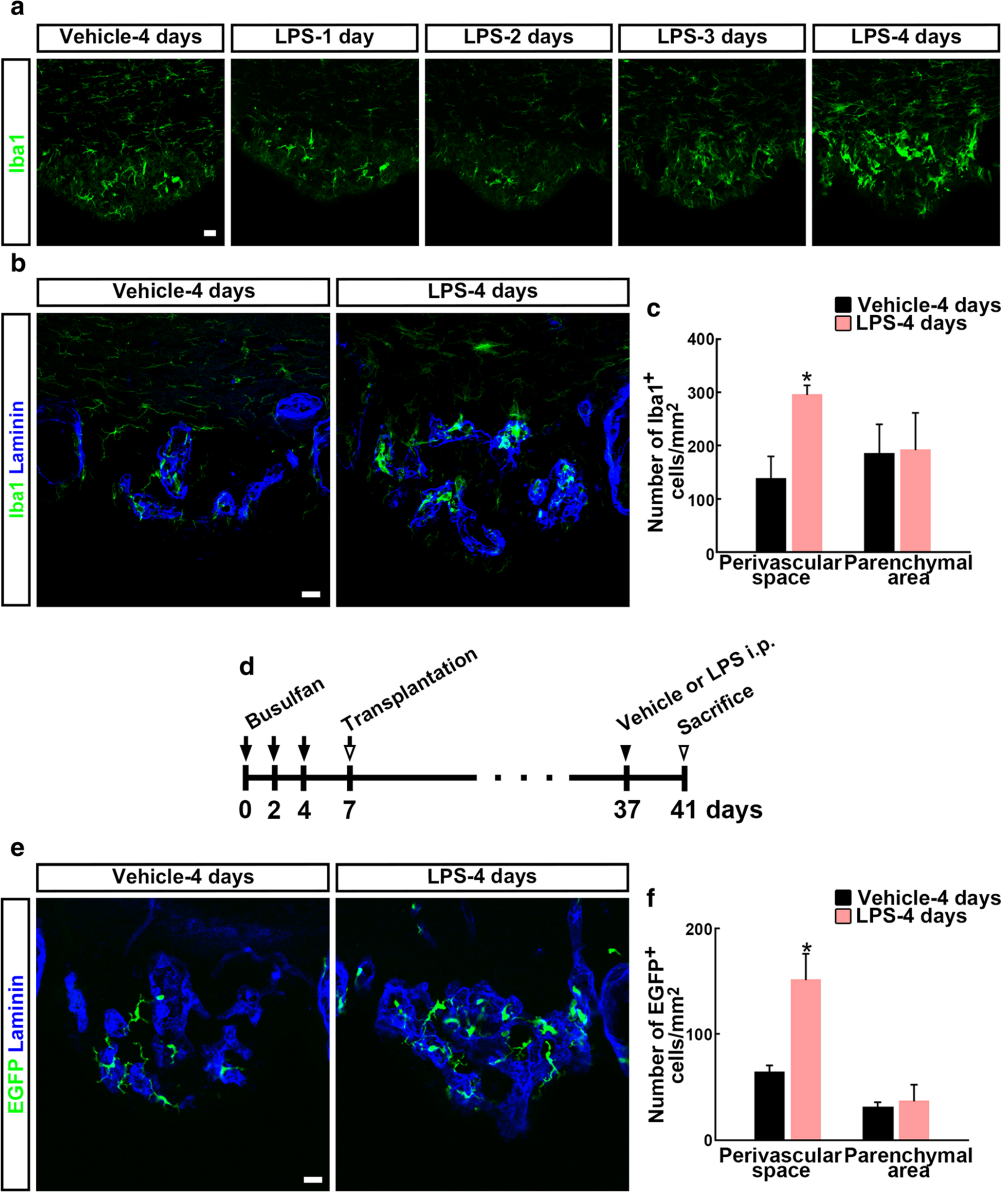
Effect of LPS injection on the number of Iba1^+^ cells and bone marrow-derived cells in the SFO. Cryosections were immunostained with antibodies against the myeloid lineage cell marker Iba1 and the outer basement membrane marker laminin-111. **a** We sacrificed mice every day after LPS injection and found an increase of Iba1^+^ cells 4 days after LPS injection. **b** Iba1^+^ cells were often seen in the SFO perivascular space of vehicle-treated mice. The number of these cells was increased inside or spanning the laminin-111^+^ outer basement membrane 4 days after LPS injection. **c** Quantitative analysis revealing the effects of LPS on the number of Iba1^+^ cells in the SFO of adult mice. *N* = 4. *P < 0.05, determined by Student’s *t* test. **d** Experimental design of bone marrow-derived cell transplantation. Mice were injected with busulfan three times (every second day; solid arrows) for immunosuppression prior to bone marrow-derived cell transplantation (open arrow). One month later, busulfan-treated chimeras were injected with vehicle or LPS (solid arrowhead) and sacrificed 4 days later (open arrowhead). **e** EGFP^+^ cells were often seen in the SFO of vehicle-treated mice. LPS injection increased the number of these cells inside or spanning the laminin-111^+^ outer basement membrane. **f** Quantitative analysis revealing the effects of LPS on the number of EGFP^+^ cells in the SFO of adult mice. *N* = 4. *P < 0.05, determined by Student’s *t* test. LPS (vehicle)-1, − 2, − 3, or − 4 days, 1, 2, 3, or 4 days after LPS (vehicle) injection; Laminin, laminin-111. Scale bars are 10 μm

To explore the effect of the first LPS injection on the SFO perivascular macrophages in endotoxin-tolerant mice, we quantified the number of Iba1^+^ cells in the SFO 4 days after the first LPS injection. There was no increase in the number of Iba1^+^ cells for at least the first 2 days after LPS injection (Fig. 4a). However, we observed a significant increase in the total number of SFO Iba1^+^ perivascular macrophages 4 days after LPS injection (139.1 ± 40.3 cells in vehicle-pre-injected mice and 296.5 ± 16.4 cells in LPS-pre-injected mice, *F*_3,3_ = 7.143, *P* = 0.023) (Fig. 4a–c). The number of Iba1^+^ cells in the parenchymal area of the SFO was not significantly different between LPS- and vehicle-injected mice.

Next, we examined whether the additional perivascular macrophages were bone marrow-derived cells using bone marrow chimeras. As described in the “Methods” section, mice were immunosuppressed by busulfan treatment, transplanted with bone marrow-derived cells from transgenic mice expressing EGFP ubiquitously, and then injected with vehicle or LPS (Fig. 4d). The number of SFO EGFP^+^ cells increased 4 days after a single LPS injection (Fig. 4e). In the perivascular space, the number of EGFP^+^ cells (64.6 ± 5.6 cells in vehicle-injected mice and 151.7 ± 24.0 cells in LPS-injected mice, *F*_3,3_ = 9.261, *P* = 0.012) significantly increased after the single LPS injection (Fig. 4f). On the other hand, we did not observe a significant change in the number of parenchymal EGFP^+^ cells. These data indicate that the SFO perivascular bone marrow-derived cells increased in number as a result of the first LPS injection.

### IL-1β expression is attenuated in SFO perivascular macrophages after the second LPS injection in endotoxin-tolerant mice

**Fig. 5:**
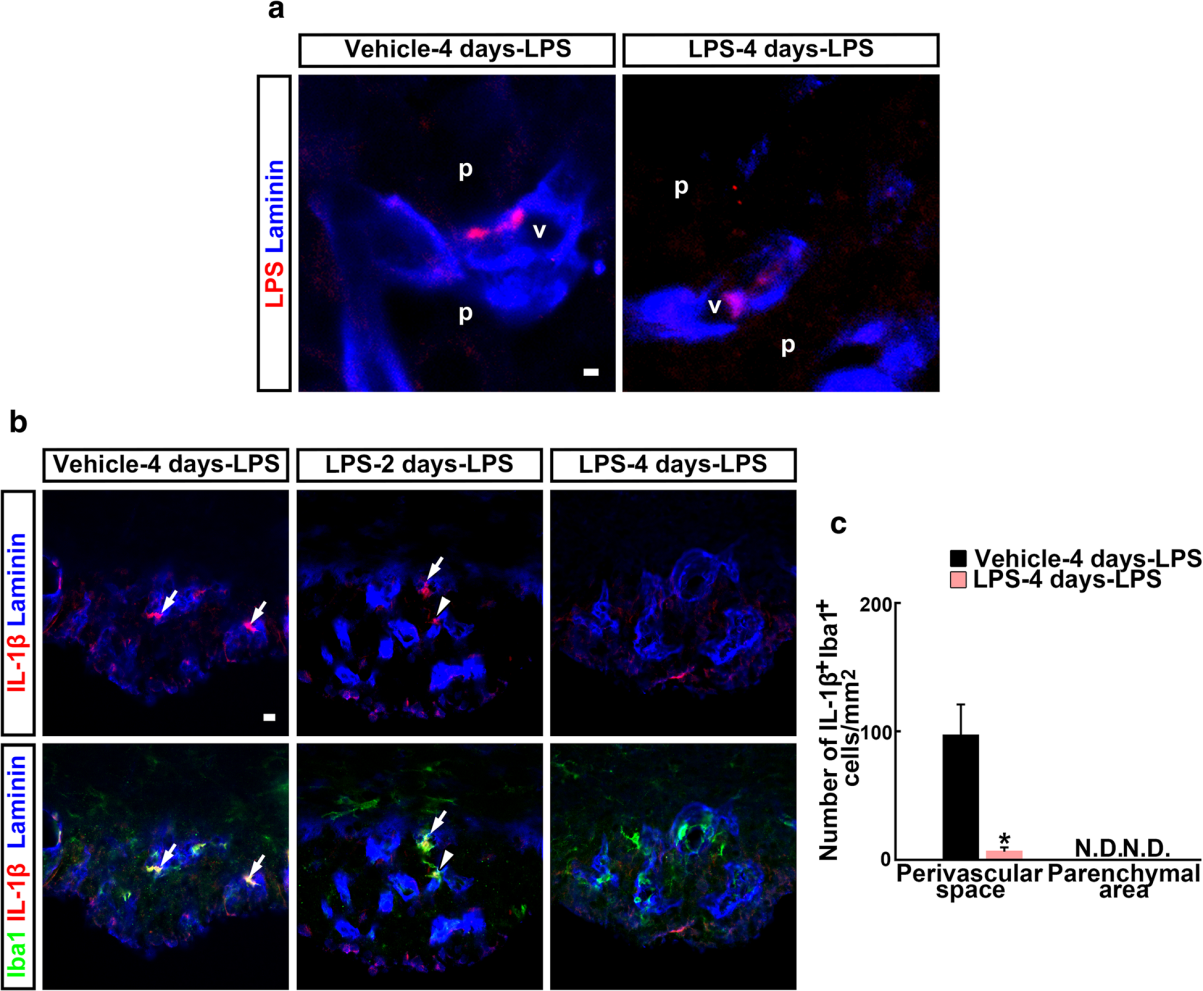
Effect of the second LPS injection on IL-1β immunoreactivity in the SFO. Cryosections were immunostained with antibodies against LPS, the myeloid lineage cell marker Iba1, and the outer basement membrane marker laminin-111. **a** Immunoreactivity of LPS was found in the SFO perivascular space 1 h after the second dose of LPS injection of vehicle- and LPS-pre-injected mice. **b** Immunohistochemistry showed that IL-1β expression was predominantly observed in the Iba1^+^ perivascular macrophages inside (arrows) or spanning (arrowheads) the laminin-111^+^ outer basement membrane following the second LPS injection 4 days after the first vehicle injection (vehicle-4 days-LPS) and 2 days after the first LPS injection (LPS-2 days-LPS). In contrast, following the second LPS injection 4 days after the first LPS injection (LPS-4 days-LPS), IL-1β immunoreactivity was rarely found in the Iba1^+^ perivascular macrophages of mice injected with LPS for the first dose. **c** Quantitative analysis revealing the effects of second LPS injection 4 days after the first vehicle or LPS injection on the number of IL-1β^+^ Iba1^+^ cells in the SFO of adult mice. *N* = 4. *P < 0.05, determined by Student’s *t* test. Laminin, laminin-111; N.D., not detected; p, parenchymal area; v, vasculature. Scale bars are 10 μm

Finally, we examined the effect of the second LPS injection on IL-1β expression in the SFO of endotoxin-tolerant mice. LPS immunoreactivity was observed in the SFO perivascular space after the second LPS injection given 4 days after the first injection (LPS-4 days-LPS) (Fig. 5a). In the SFO Iba1^+^ perivascular macrophages, IL-1β immunoreactivity was observed 1 h after the second LPS injection in vehicle-pre-injected mice (vehicle-4 days-LPS) and in LPS-2 days-LPS mice, whereas it was rarely observed in LPS-4 days-LPS mice (Fig. 5b). The number of SFO IL-1β^+^ Iba1^+^ perivascular macrophages significantly decreased in LPS-4 days-LPS mice as compared to that in LPS-4 days-vehicle mice (97.5 ± 23.3 cells in LPS-4 days-vehicle mice and 7.2 ± 2.6 cells in LPS-4 days-LPS mice, *F*_3,3_ = 100.23, *P* = 0.030) (Fig. 5c). These data indicate that perivascular macrophages ceased to produce IL-β in the SFO concomitantly with the development of endotoxin tolerance, even though circulating LPS entered the SFO perivascular space after the second LPS injection.

## Discussion

**Fig. 6:**
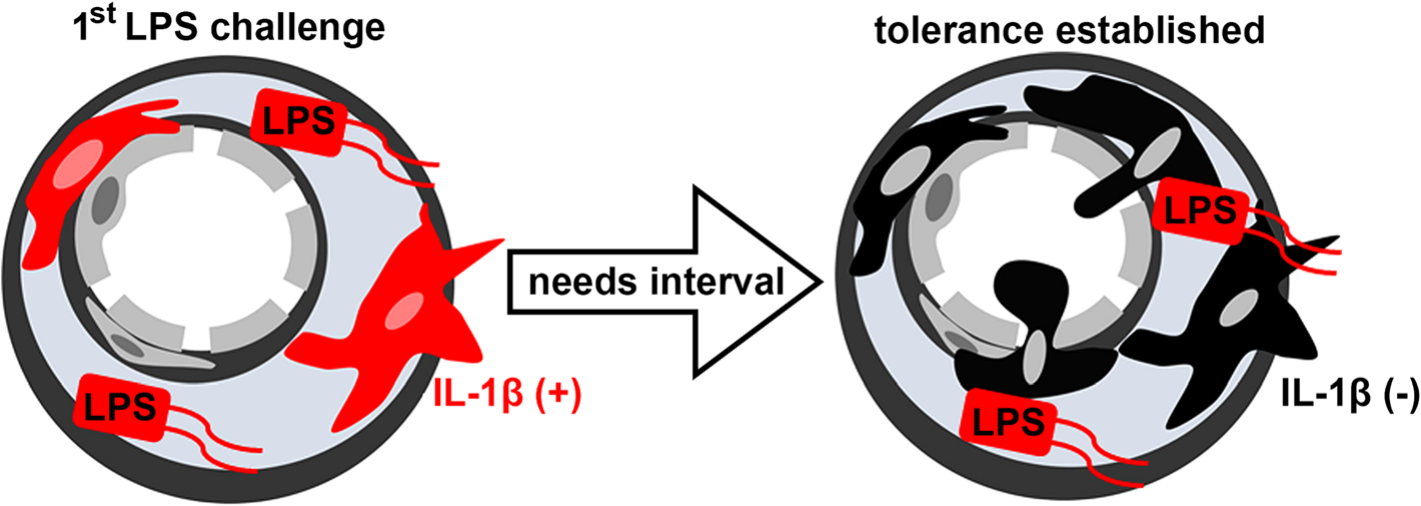
A schematic diagram. SFO Iba1^+^ perivascular macrophages expressed IL-1β 1 h after the first dose of peripheral LPS injection (first LPS challenge). Four days after the first injection, mice became endotoxin-tolerant. Bone marrow-derived perivascular macrophages accumulated in the SFO and did not express IL-1β after the second dose of LPS (tolerance established)

The aim of this study was to explore the role of the SFO perivascular macrophages for IL-1β production in LPS-non-tolerant and LPS-tolerant mice. Perivascular macrophages that localized outside the brain parenchyma expressed IL-1β in the SFO after the first LPS injection. Circulating LPS did not diffuse into the SFO parenchyma. Perivascular macrophages were Iba1^+^, phagocytic, and bone marrow-derived cells. Macrophage deprivation results in the attenuation of LPS-induced IL-1β expression in the SFO. The SFO perivascular macrophages rarely expressed IL-1β after the second LPS injection in endotoxin-tolerant mice, although bone marrow-derived SFO macrophages increased and peripherally injected LPS reached to the SFO perivascular space. A schematic diagram summarizing these findings is provided in Fig. 6. These results suggest that the SFO ceases IL-1β production when animals become endotoxin-tolerant, because bone marrow-derived perivascular macrophages become hyporesponsive to LPS.

Perivascular macrophages were the initial source of LPS-induced IL-1β in the SFO. Macrophages around the SFO vasculature were first proposed as IL-1β source cells [6], but why they rapidly express IL-1β, and microglia do not, is unknown. In the present study, we distinguished the perivascular space and parenchyma in the SFO by visualizing the outer basement membrane and clarified that perivascular macrophages localized mainly in the perivascular space, i.e., outside the brain parenchyma. Moreover, clodronate liposomes depleted perivascular macrophages and attenuated LPS-induced IL-1β production. Peripherally injected LPS localized in the perivascular space in the SFO. Since intracerebroventricular injection of LPS induces neuronal apoptosis [26], it is reasonable that the penetration of circulating LPS should be restricted in the SFO, which possesses neurons in its parenchyma like other brain regions. In the SFO, CD45^+^ immune cells and GFAP^+^ astrocytes express a LPS receptor, toll-like receptor 4 [27]. Upregulation of IL-1β after LPS injection was mainly observed in Iba1^+^ perivascular macrophages, whereas IL-1β was constantly expressed in parenchymal cells, which mainly consist of GFAP^+^ astrocytes. These observations suggest that perivascular macrophages are responsible for IL-1β production in response to circulating LPS. Together, these data indicate that the localization of IL-1β-producing cells enables the SFO to sense circulating LPS and thus to avoid severe neuron damage.

Another key finding is that when mice become endotoxin-tolerant, perivascular macrophages scarcely produced IL-1β in the SFO, although peripherally injected LPS and an increased number of bone marrow-derived cells were observed in the perivascular space. Endotoxin tolerance is not only known as animal hyporesponsiveness, but also known as macrophage hyporesponsiveness to restimulation by endotoxin (for a review, see [28]). LPS-pre-exposed, endotoxin-tolerant murine macrophages and human leukocytes exhibit suppression of LPS-induced expression of mRNA for IL-1β in vitro [29, 30]. These data suggest that the SFO perivascular macrophages become tolerant by the first LPS injection and may contribute to the development of behavioral endotoxin tolerance. However, the mechanistic details require further investigation, and a direct role for accumulated bone marrow-derived cells in behavioral endotoxin tolerance is yet to be confirmed. Future studies will need to focus on the phenotype of SFO perivascular macrophages to elucidate their temporal changes.

We observed that some perivascular macrophages spanned the outer basement membrane. Similar to LPS, IL-1β is unlikely to diffuse into the SFO parenchyma since it is a large (MW: 17,300) hydrophilic protein. Macrophages that span the outer basement membrane may deliver IL-1β to the SFO parenchyma. Once it enters the brain parenchyma, IL-1β can induce IL-1β production by adjacent microglia [31]. Parenchymal microglia express IL-1β in the SFO at later times after LPS injection [32]. In addition, IL-1β induces a transient depolarization in a subpopulation of isolated SFO neurons [33]. These data suggest that IL-1β propagates its own synthesis to stimulate neurons. Another possibility is that diffusible second messengers are produced downstream of IL-1β. Although IL-1 receptor-expressing vessels exist throughout the brain, especially strong immunoreactivity for the IL-1 receptor is observed in the vasculature of the SFO [34]. IL-1β is able to induce cyclooxygenase-2 activity and the production of prostaglandin E2, which crosses the BBB [35]. Local communication between perivascular macrophages and endothelial cells may thus occur in the SFO perivascular space.

In the present study, we only used male adult mice to simplify the experiment. Because females and offspring are also exposed to endotoxins, it remains for future studies to investigate the effect of LPS on female and developing brains.

## Conclusions

SFO perivascular macrophages were the IL-1β source cells in LPS-non-tolerant mice. In contrast, they cease expressing IL-1β in tolerant mice, although bone marrow-derived perivascular macrophages increase in number and peripherally injected LPS reach to the SFO perivascular space. The SFO perivascular macrophages may thus have a role in endotoxin tolerance.

## Additional file

Additional file 1: **Figure S1.** Engraftment of bone marrow-derived cells into the SFO. **Figure S2.** Specificity controls for IL-1β antibody. **Figure S3.** Characterization of the SFO perivascular Iba1^+^ cells. **Figure S4.** Localization of peripherally injected control liposomes in the SFO. (PDF 852 kb)

## Abbreviations

BBB: Blood-brain barrier
EGFP: Enhanced green fluorescent protein
GFAP: Glial fibrillary acidic protein
Iba1: Ionized calcium-binding adaptor molecule 1
IL-1β: Interleukin-1β
LPS: Lipopolysaccharides
PBS: Phosphate-buffered saline
SFO: Subfornical organ
