# Supplementary material for: Responses of perivascular macrophages to circulating lipopolysaccharides in the subfornical organ with special reference to endotoxin tolerance
Source: J Neuroinflammation. 2019 Feb 14;16:39. doi: 10.1186/s12974-019-1431-6 (PMC6375194; doi:10.1186/s12974-019-1431-6)
Supplement: Supplementary file 1 — Figure S1. Engraftment of bone marrow-derived cells into the SFO. Figure S2. Specificity controls for IL-1β antibody. Figure S3. Characterization of the SFO perivascular Iba1+ cells. Figure S4. Localization of peripherally injected control liposomes in the SFO. (PDF 852 kb) [file 12974_2019_1431_MOESM1_ESM.pdf]

Journal of Neuroinflammation

Additional file 1

Responses of perivascular macrophages to circulating lipopolysaccharides in the subfornical organ with special reference to endotoxin tolerance

Shoko Morita-Takemura・Kazuki Nakahara・Sanae Hasegawa-Ishii・Ayami Isonishi・Kouko Tatsumi・Hiroaki Okuda・Tatsuhide Tanaka・Masahiro Kitabatake・Toshihiro Ito・Akio Wanaka

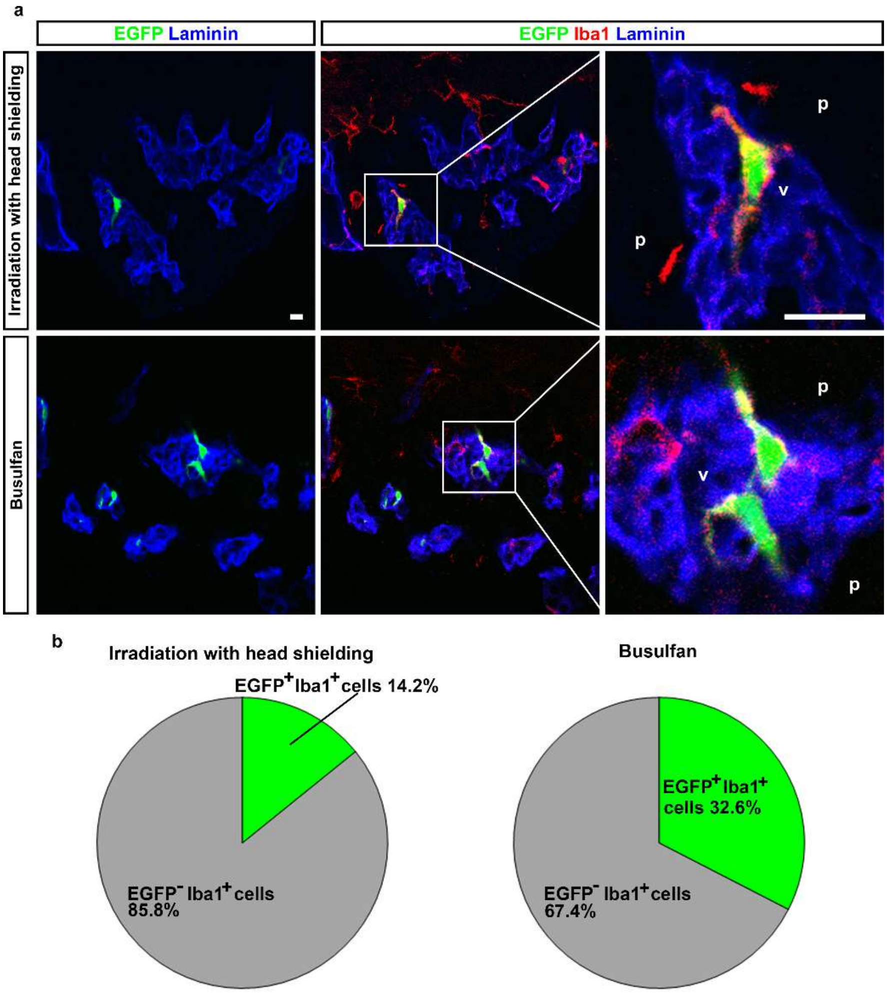

Supplementary Fig. 1. Engraftment of bone marrow-derived cells into the SFO. Cryosections were immunostained with antibodies against the myeloid lineage cell marker Iba1 and the outer basement membrane marker laminin-111. (a) Immunohistochemistry shows $EGFP^{+}$ $Iba1^{+}$ cells in the SFO of irradiated chimeric mice with head shield 4 weeks after reconstitution (upper) and that of busulfan-treated chimeric mice 4 weeks after reconstitution (lower). (b) Quantification of results from a. Data were obtained from 4-5 animals (37-46 sections) and 289-470 $Iba1^{+}$ perivascular macrophages were examined. *Laminin* laminin-111, *p* parenchymal area, *v* vasculature. Scale bars are 10 μm.

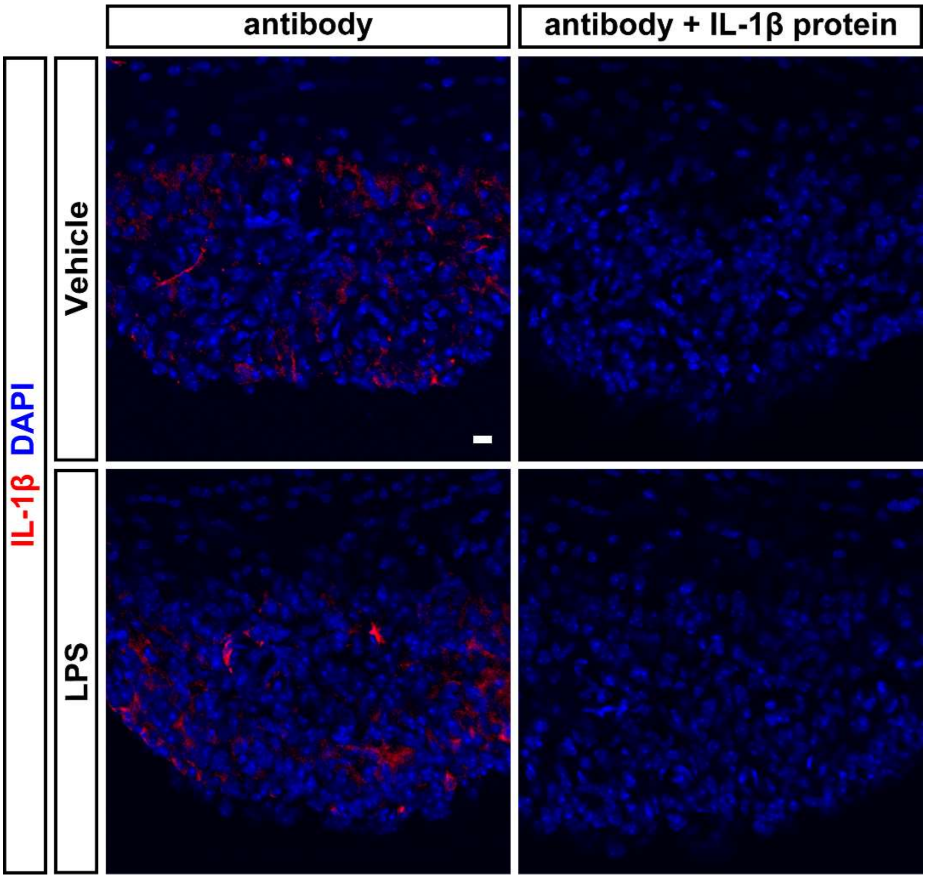

Supplementary Fig. 2. Specificity controls for IL-1β antibody. (upper) Immunohistochemistry was performed on mouse brain sections with anti-IL-1β antibody. (b) Anti-IL-1β antibody was pre-adsorbed with IL-1β recombinant protein and used for immunohistochemistry preadsorption control. The IL-1β staining was significantly diminished or abolished when the diluted primary antibody was preincubated with the IL-1β recombinant protein. (lower) The IL-1β staining was also abolished by antibody pre-incubation with the IL-1β recombinant protein in the SFO of LPS-injected mice. Nuclei were counterstained with 4,6-diamidino-2-phenylindole. Scale bar is 10 μm.

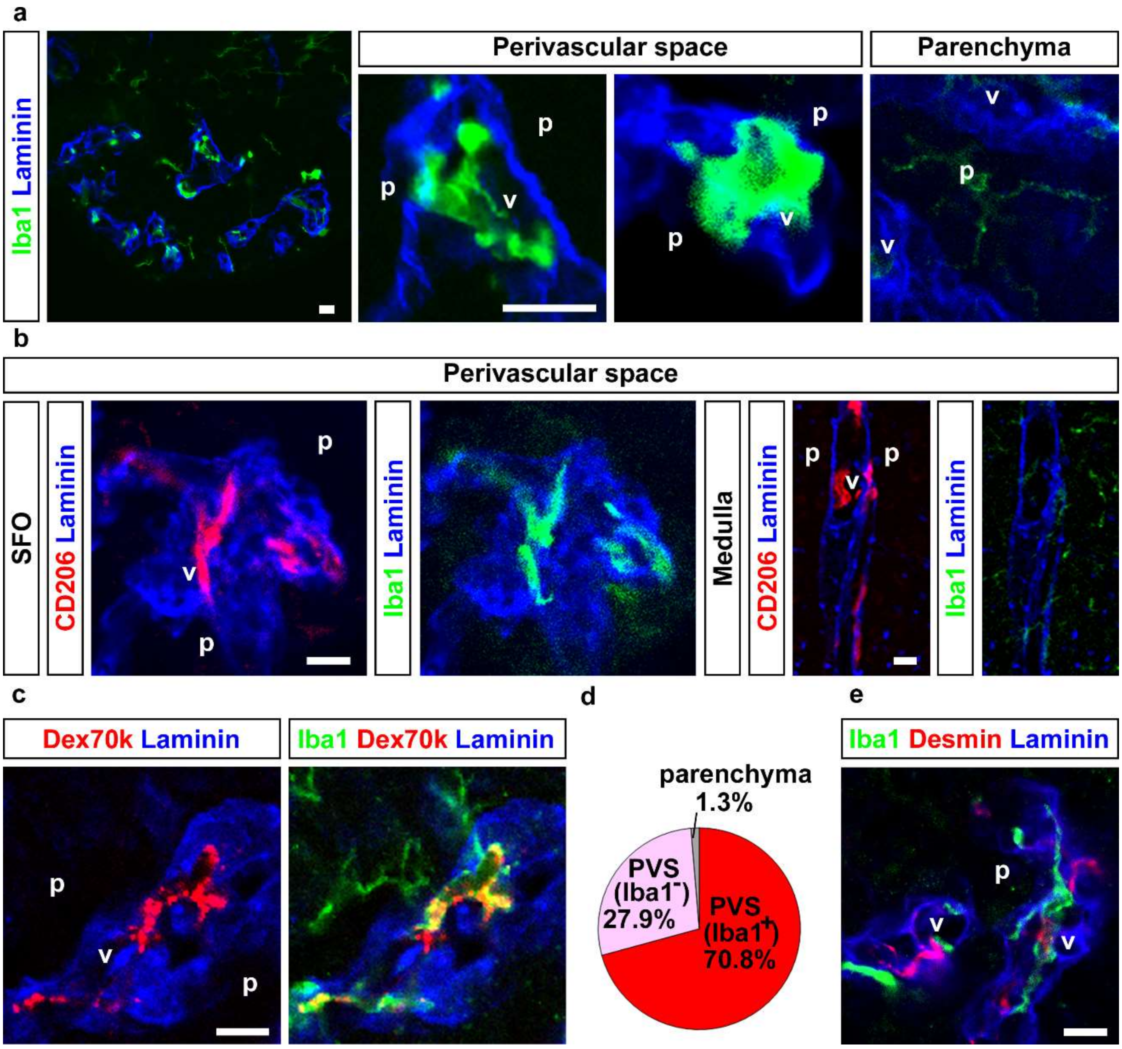

Supplementary Fig. 3. Characterization of the SFO perivascular Iba1⁺ cells. Cryosections were immunostained with antibodies against the myeloid lineage cell marker Iba1, the outer basement membrane marker laminin-111, the mannose receptor CD206, and the pericyte marker desmin. (a) Immunohistochemistry revealed that the Iba1⁺ cells were widely distributed in the SFO. Perivascular Iba1⁺ cells were seen inside, or sometimes spanning, the laminin-111⁺ outer basement membrane, and were strongly immunoreactive for Iba1 and exhibited elongated or ameboid-like morphology. Microglia were seen outside the laminin-111⁺ outer basement membrane, were immunoreactive for Iba1, and exhibited ramified morphology. (b) Both the SFO perivascular Iba1⁺ cells and typical perivascular macrophages were CD206⁺, but typical perivascular macrophages were weakly

immunoreactive for Iba1. (c) The SFO perivascular Iba1$^+$ cells were able to phagocytose circulating dextran. (d) Quantification of results from c. Data were obtained from 5 animals (48 sections) and 383 Dextran 70,000$^+$ spots were examined. (e) The SFO perivascular Iba1$^+$ cells were negative for the pericyte marker desmin. *Dex70k* Dextran 70,000, *Laminin* laminin-111, *p* parenchymal area, *PVS* perivascular space, *v* vasculature. Scale bars are 10 μm.

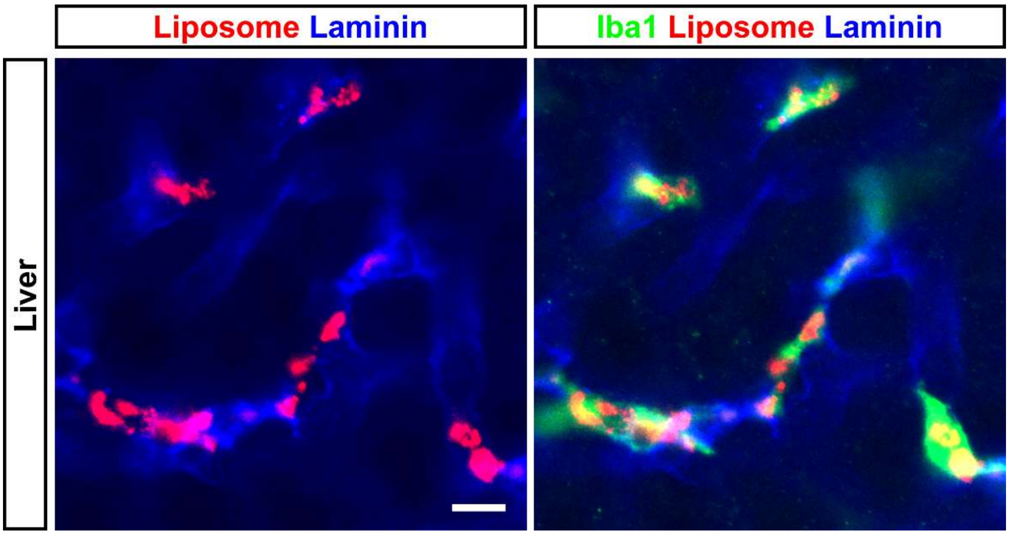

Supplementary Fig. 4. Localization of peripherally injected control liposomes in the SFO. Cryosections were immunostained with antibodies against the myeloid lineage cell marker Iba1 and the outer basement membrane marker laminin-111. DiI-labeled control liposomes were found in the liver and co-localized with Iba1 immunoreactivity. *Laminin* laminin-111. Scale bar is 10 μm.
